# Supplementary material for: Cloud BioLinux: pre-configured and on-demand bioinformatics computing for the genomics community
Source: BMC Bioinformatics. 2012 Mar 19;13:42. doi: 10.1186/1471-2105-13-42 (PMC3372431; doi:10.1186/1471-2105-13-42)
Supplement: Additional file 1 — Supplementary 1 Cloud BioLinux software documentation in the form of a mini, self-contained website. Users need to download and uncompress the .zip file, and open through a web browser the "index.html" file available on the main directory. (ZIP 1823 kb). [file 1471-2105-13-42-S1.ZIP › Cloud-BioLinux-Package-Documentation/Phylogeny.html]

Bio-Linux Software Documentation Pages

Back to search form

### Phylogeny

|  |  |
| --- | --- |
| Category | Applications |
| Phylogeny | jMOTU    phylip    FastTree    mothur    evolver |
| SubCategory | Applications |
| Bayesian | migrate    mrbayes    mrbayes-multi    qiime |
| Clustering | dotur    qiime |
| Consensus | consense |
| Continuous\_characters | contml    contrast |
| Discrete\_characters | clique    dollop    dolpenny    factor    mix    penny |
| Distance\_matrix | arb    fitch    kitsch    mothur    neighbor |
| Gene\_frequencies | gendist |
| Maximum\_likelihood | arb    baseml    basemlg    codeml    fastdnaml    mcmctree    pamp    Phyml    proml    PROmlk    restml    tree-puzzle    yn00 |
| Maximum\_parsimony | dnamove    pars |
| Misc | bioperl    extract\_from\_pdb    mesquite    pedro    qiime    seqsplit |
| Molecular\_sequence | clustalw    clustalx    distmat    dnacomp    dnadist    dnainvar    dnaml    dnamlk    dnapars    dnapenny    protdist    protpars    qiime    seqboot |
| Tree\_drawing | ape    arb    atv    dendroscope    njplot    qiime    retree    splitstree    treedist    treeview    xsplits |
